# Postoperative Pain Management among Registered Nurses in a Vietnamese Hospital

**DOI:** 10.1155/2020/6829153

**Published:** 2020-08-11

**Authors:** Phuong Hoang Vu, Duc Viet Tran, Yen Thi Le, Ha Thi Thu Do, Sao Thi Vu, Huong Thanh Dinh, Tu Huu Nguyen

**Affiliations:** ^1^Hanoi Medical University, Hanoi, Vietnam; ^2^Hanoi Medical University Hospital, Hanoi, Vietnam

## Abstract

This study examined the postoperative pain management practices among registered nurses in an urban hospital in Vietnam. Data of 90 nurses about postoperative pain management practices and pain management at the department were collected. Results indicated that 83.3% of nurses reported that they regularly assessed the degree of pain for postoperative patients. Only 32.2% used assessment tools such as the numeric rating scale to measure pain. Experience in pain management and having guidelines in the department were associated with a higher score in pain management practice. Findings suggested that facilitating the use of pain instruments and developing pain management guidelines should be prioritized.

## 1. Introduction

Postoperative pain management is a great issue although substantial progress in analgesic technologies and clinical guidelines has been made in recent years [1]. Previous reports revealed a large proportion of patients experiencing mild-to-severe pain after surgery [2], as well as pain-related consequences [3]. Health professionals play a major role in poor postoperative pain management. Many studies underlined that insufficient pain management education and negative attitude toward patients who sought medical treatment for their pain were significant barriers [4–6]. Moreover, the culture of the hospital and the context of pain have been found as contributors to the pain treatment failure [7–9].

Among health professionals, nurses are key personnel to support patients experiencing pain in both assessment and treatment, given their large amount of time to spend with the patients [10]. Nonetheless, prior literature points out the lack of knowledge in pain management among nurses [11, 12]. Moreover, in the clinical setting, nurses' knowledge and practices are not always consistent [13, 14]. They seem to normalize pain after surgery as an acceptable condition that patients have to suffer, leading to a lack of effort or priority in relieving the pain [13, 15]. This causes a huge gap between nurses' perception and implementation in postoperative pain management, which possibly poses a great challenge to effective pain management in the hospital. Therefore, understanding how nurses practice postoperative management as well as identify factors related to these practices are critically important. This study aimed to examine the postoperative pain management practices among registered nurses in an urban hospital in Vietnam.

## 2. Methods

### 2.1. Study Design and Sample

This is a descriptive cross-sectional study that was conducted in May 2019 at the Hanoi Medical University Hospital, Hanoi, Vietnam, as a baseline of an intervention. Nurses were recruited from eight departments who carried out minor and major surgeries in the hospital including Otorhinolaryngology (ORL), Plastic Surgery, Oncology, Odonto-Stomatology, Outpatient, Trauma and Orthopaedics, General Surgery A (Neuro and Spine Surgery), and General Surgery B (Gastro-Intestinal Surgery, Hepato-Biliary Surgery, and Urology Surgery). They were included in the study if they (1) were aged 18 years or above; (2) worked in the selected departments of the hospital for at least 6 months, and (3) agreed to participate in the study. A total of 90 nurses were invited, and all of them accepted to be involved in the study (response rate 100%). The protocol of this study was approved by the Institutional Review Board of Hanoi Medical University (code: 165/QD-DHYHN) and the leaderboard of the hospital.

### 2.2. Data Collection and Measurement

Data collectors were members of the research team who were junior physicians working at the hospital. They were trained intensively with two training sessions in order to collect data consistently and with the highest quality. Training contents included communication skills, interview skills, and questionnaire. The questionnaire was developed and piloted with ten nurses by the data collectors to ensure the language and logical orders of items. The revised questionnaire was then approved by principal investigators and the hospital's leaderboard.

Face-to-face interviews were performed using a finalized structured questionnaire. Each interview lasted from 15 to 20 minutes. Nurses were first invited to a private room at their department to ensure their privacy. After being informed of a brief introduction of the study, they were asked to give their signature for the written informed consent forms. Participants were then asked to report the following information: demographic characteristics (including age, gender, education, and previous training in pain management), postoperative pain management practices, and pain management protocol at the department.

#### 2.2.1. Postoperative Pain Management Practices

To measure the practices on pain management among nurses, we asked them to report what types of pain they had care experience (acute/chronic/both/none), types of patients receiving the assessment, time for pain assessment, and their knowledge about basic side effects of pain relief medications. Then, they were asked to report whether they regularly performed pain assessment (yes = 1 point/no = 0 point) and the frequency of (1) side effects monitoring; (2) pain level assessment when changing patient's position; (3) patients' self-reported pain information collection; and (4) care plan change based on results of pain assessment, with four options: none (0 point)/rarely (1 point)/usually (2 points)/always (3 points). The practice score was a sum of five questions, resulting in a total score ranging from 0 to 13, in which a higher score indicated a higher level of practice.

#### 2.2.2. Pain Management at the Department

Participants were asked to report whether (1) pain management service, (2) pain management guidelines, and (3) pain medication's side effects management guidelines were available at the department. We assumed that nurses working in a similar department might receive similar pain management training and perceive similar barriers regarding postoperative pain management. Moreover, they were asked to report whether the prescription of pain relief medication was based on pain assessment at the department or not, as well as available pain relief medications and barriers of pain management implementation at the department.

### 2.3. Statistical Analysis

Data analysis was performed using the Stata software version 15.0. Descriptive statistics analysis was conducted including mean and standard deviation for continuous variables and frequency and percentage for categorical variables. The mixed-effect linear regression model was employed to determine the associated factors with the practice score via controlling the cluster effect within the department. The dependent variable was practice scores, while the independent variables were age, gender, education, types of pain experienced in care, having training about pain management, availability of pain management service/pain management guideline/side effects management guidelines, and barriers in pain management implementation. The regression coefficient, *p* value, and 95% confidence interval (CI) were presented. A *p* value of less than 0.05 was recognized to detect statistical significance.

## 3. Results

Among 90 nurses participating in the study, most of them were female (75.6%). The mean age was 30.7 (SD = 3.9) years old. The majority of nurses had college or vocational training degrees (51.1%) and did not have training about pain management previously (72.2%) (Table 1).

Regarding postoperative pain management practices, Table 2 shows that eleven nurses did not care for pain patients (12.2%) previously. The majority of nurses assessed pain for all patients (33.3%), based on physicians' orders (27.8%), and when patients reported pain (60.0%). Only three nurses knew all of the side effects of pain relief medications (3.3%).

Seventy-five nurses (83.3%) reported that they regularly assessed the degree of pain for postoperative patients. Only 32.2% used assessment tools such as the numeric rating scale to measure pain. Most nurses usually/always monitored side effects (76.7%), assessed pain when changing the patient's position (80.0%) and patients' self-reported pain level, and changed the care plan due to pain assessment (86.7%). Overall, the mean practice score was 8.4 (SD = 1.7).

In terms of pain management at the department, Table 3 shows that only 67.0%, 33.0%, and 45.6% reported that their department had pain management service, pain management guidelines, and pain medication's side effects management guidelines, respectively. 64.4% reported that physicians in their department prescribed pain relief medication based on pain assessment. Paracetamol was the most dominant medication (97.8%), following by morphine (48.9%) and anti-inflammation drugs (36.7%).

**Figure 1:**
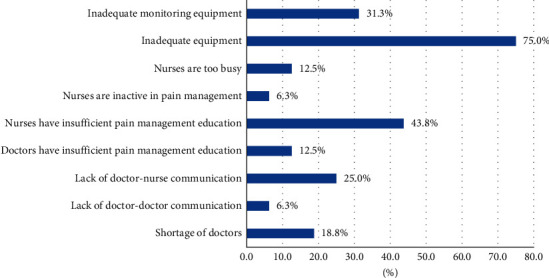
Barriers of pain management implementation at the department (*n* = 16).

Among 16 nurses reporting barriers of pain management implementation at the department, the major barrier was inadequate equipment (75.0%), followed by insufficient education among nurses (43.8%) and inadequate pain monitoring equipment (31.3%) (Figure 1).

Table 4 shows that nurses experienced in caring for both patients with acute and chronic pain had a higher practice score than those without experience (Coef. = 0.46, 95% CI = 0.04; 0.88). Meanwhile, the practice score of those reporting that their department did not have guidelines for side effect management had 1.50 points lower than those having the guidelines in their department (Coef. = −1.50, 95% CI = −2.25; −0.76).

## 4. Discussion

Our study partly filled the knowledge gap about the postoperative pain management practice among nurses in Vietnam. Our findings indicated a moderate level of practice in pain management after surgery. Moreover, we pointed out potential barriers as well as associated factors for pain management practice, which are helpful for designing further interventions to improve this issue in the hospital setting.

In this study, we found that the majority of nurses reported performing pain assessments regularly, as well as other tasks of pain management such as monitoring side effects, assessing pain degrees when changing the patient's position, or changing the care plan based on results of pain assessment. However, we observed that most of the nurses performed these tasks when patients reported pain rather than carrying out these works routinely as pain management. Indeed, after operations, patients are more likely to suffer from temporary cognitive impairment [16]; thus, it is difficult for them to communicate verbally to reflect their needs in pain relief [16]. A previous study showed that patients could only express a few details about their degree of pain [10], and indirect questions would reduce the possibility to evaluate accurately patients' pain condition [13]. We believed that high workloads might be an attribute to this phenomenon since nurses have to pay attention or prioritize other activities [16]. Notably, our findings revealed that only one-third of nurses used assessment tools such as the numeric rating scale to measure pain, while most of them only asked patients a simple question to evaluate pain conditions. Schafheutle et al. and Dihle et al. in their studies suggested that nurses frequently relied on their own judgments to evaluate patients' pain conditions [13, 17]. The percentage of nurses not using the assessment tool in our study was higher than that in a prior study in Sweden [18] but lower than findings in other settings [19, 20]. Utilizing pain instruments is particularly important to improve nurse-patient communications by sharing the same language [21], which enables patients to express their pain circumstances as well as needs of pain relief. Moreover, nurses can use this measurement to evaluate the effectiveness of pain relief therapies [13, 17]. Collectively, these are a huge gap in pain assessment practices among urban nurses. Thus, training nurses to carry out pain assessment more regularly instead of waiting for the patients' report and motivating the use of pain instruments among nurses should be prioritized in further interventions.

In the current study, we found that the pain management service or guidelines were insufficient in the hospital setting although many surgical operations had been implemented. The regression result showed that lack of guidelines such as side effects management could reduce the postoperative pain management practice among nurses. In the literature, a lack of knowledge and documents resulted in insufficient systematic performance in pain management; or in other words, routine-driven operative pain management outweighed knowledge-driven practice [10]. Unless systematic guidelines and intensive training are given to them for clinical application, this issue might pose a great challenge in the enhancement of the postoperative pain management practice among nurses.

Our study has some limitations. First, although we recruited all nurses in the selected department, our sample size was still small. The result should be carefully applied in other settings. Second, our questions were not validated but were merely rapid assessments. Further studies with validated questionnaires about postoperative pain practices should be performed. Finally, our study used a cross-sectional design, which limited our ability to draw the causal conclusions for the associations between practice score and other factors.

## 5. Conclusion

This study underlined a moderate level of postoperative pain management practice among nurses. Facilitating the use of pain instruments and developing pain management guideline should be prioritized to improve the nurses' practice toward postoperative pain management.

## Figures and Tables

**Table 1:** Demographic characteristics.

Characteristics	*n*	%
Department		
ORL	9	10.0
Plastic surgery	7	7.8
Oncology	21	23.3
Odonto-stomatology	7	7.8
Outpatient clinic	14	15.6
Trauma and orthopaedics	6	6.7
General surgery A	6	6.7
General surgery B	20	22.2

Gender		
Male	22	24.4
Female	68	75.6

Education		
High school	2	2.2
College or vocational training	46	51.1
University or above	42	46.7

Having training about pain management		
Yes	25	27.8
No	65	72.2

	**Mean**	**SD**

Age	30.7	3.9

**Table 2:** Pain management practices among nurses.

Characteristics	*n*	%
Types of pain experienced in care		
Acute pain	17	18.9
Chronic pain	5	5.6
Both	57	63.3
None	11	12.2

Types of patients receiving assessment		
Based on physicians' order	25	27.8
Based on self-identified	18	20.0
All patients	30	33.3
Patients who compulsorily required pain assessment	14	15.6

Time for pain assessment		
Consistent time interval	28	31.1
When patients report pain	54	60.0
During monitoring phase	16	22.2
During care phase	34	37.8
Others	2	2.2

Knowing basic side effects of pain relief medications		
No	1	1.1
Some side effects	41	45.6
Most side effects	45	50.0
All side effects	3	3.3
Regular pain assessment	75	83.3

Method to measure pain		
Ask patients a simple question	59	65.6
Using assessment tool (numeric rating scale)	29	32.2
Others	3	3.3

Frequency of monitoring side effects		
None	3	3.3
Rarely	18	20.0
Usually	63	70.0
Always	6	6.7

Frequency of pain assessment when changing patient's position		
None	6	6.7
Rarely	12	13.3
Usually	58	64.4
Always	14	15.6

Frequency of collecting patients' self-reported pain information		
None	0	0.0
Rarely	14	15.6
Usually	67	74.4
Always	9	10.0

Frequency of care plan change due to pain assessment		
None	4	4.4
Rarely	8	8.9
Usually	68	75.6
Always	10	11.1

	**Mean**	**SD**

Practice score (0–13)	8.4	1.7

**Table 3:** Pain management at the department.

Characteristics	*n*	%
Availability in the department		
Pain management service	59	67.0
Pain management guideline	30	33.3
Pain medication's side effects management guideline	41	45.6

Prescription of pain relief medication based on pain assessment		
Yes	58	64.4
No	23	25.6
Don't know	9	10.0

Available pain relief medications		
Paracetamol	88	97.8
Anti-inflammation	33	36.7
Morphine	44	48.9
Anesthesia	17	18.9
Ketamine	3	3.3
Antidepression	3	3.3
Antiseizure	2	2.2
Others	9	10.0

**Table 4:** Associated factors with practice score.

Characteristics	Coef.	*p* value	95% CI	
Age	−0.01	0.88	−0.09	0.08

Gender				
Male	Ref.			
Female	−0.17	0.78	−1.36	1.02

Education				
Less than university	Ref.			
University or above	0.23	0.48	−0.41	0.87

Types of pain experienced in care				
None	Ref.			
Acute	0.52	0.61	−1.46	2.51
Chronic	0.32	0.50	−0.59	1.22
Both	0.46	0.03	0.04	0.88

Having training about pain management				
Yes	Ref.			
No	−0.38	0.48	−1.44	0.67

Availability of pain management service				
Yes	Ref.			
No	0.10	0.68	−0.37	0.57

Availability of pain management guideline				
Yes	Ref.			
No	0.12	0.75	−0.62	0.87

Availability of side effects management guideline				
Yes	Ref.			
No	−1.50	<0.01	−2.25	−0.76

Barriers in pain management implementation				
Yes	Ref.			
No	−0.32	0.23	−0.83	0.20

## Data Availability

Requests for access to individual subject data may be made to the corresponding author through e-mail (vuhoangphuong@hmu.edu.vn).

## References

[1] Mitra S., Carlyle D., Kodumudi G., Kodumudi V., Vadivelu N. (2018). New advances in acute postoperative pain management. *Current Pain and Headache Reports*.

[2] Yang M. M. H., Hartley R. L., Leung A. A. (2019). Preoperative predictors of poor acute postoperative pain control: a systematic review and meta-analysis. *BMJ Open*.

[3] Schug S. A., Palmer G. M., Scott D. A., Halliwell R., Trinca J. (2016). Acute pain management: scientific evidence, fourth edition, 2015. *Medical Journal of Australia*.

[4] Anderson T. (2010). The politics of pain. *British Medical Journal*.

[5] Johnson D. C., Kassner C. T., Houser J., Kutner J. S. (2005). Barriers to effective symptom management in hospice. *Journal of Pain and Symptom Management*.

[6] Schug S. A. (2011). 2011 - the global year against acute pain. *Anaesthesia and Intensive Care*.

[7] Abdalrahim M. S., Majali S. A., Stomberg M. W., Bergbom I. (2011). The effect of postoperative pain management program on improving nurses’ knowledge and attitudes toward pain. *Nurse Education in Practice*.

[8] Brant J. M., Mohr C., Coombs N. C., Finn S., Wilmarth E. (2017). Nurses’ knowledge and attitudes about pain: personal and professional characteristics and patient reported pain satisfaction. *Pain Management Nursing*.

[9] Kiekkas P., Gardeli P., Bakalis N. (2015). Predictors of nurses’ knowledge and attitudes toward postoperative pain in Greece. *Pain Management Nursing*.

[10] Bach A. M., Forman A., Seibaek L. (2018). Postoperative pain management: a bedside perspective. *Pain Management Nursing*.

[11] Matthews E., Malcolm C. (2007). Nurses’ knowledge and attitudes in pain management practice. *British Journal of Nursing*.

[12] Wang H.-L., Tsai Y.-F. (2010). Nurses’ knowledge and barriers regarding pain management in intensive care units. *Journal of Clinical Nursing*.

[13] Dihle A., Bjolseth G., Helseth S. (2006). The gap between saying and doing in postoperative pain management. *Journal of Clinical Nursing*.

[14] van Dijk J. F., Schuurmans M. J., Alblas E. E., Kalkman C. J., van Wijck A. J. (2017). Postoperative pain: knowledge and beliefs of patients and nurses. *Journal of Clinical Nursing*.

[15] Yang Y. E., Xiong C., Xia L. (2020). Consistency of postoperative pain assessments between nurses and patients undergoing enhanced recovery after gynaecological surgery. *Journal of Clinical Nursing*.

[16] Manias E., Bucknall T., Botti M. (2005). Nurses’ strategies for managing pain in the postoperative setting. *Pain Management Nursing*.

[17] Schafheutle E. I., Cantrill J. A., Noyce P. R. (2001). Why is pain management suboptimal on surgical wards?. *Journal of Advanced Nursing*.

[18] Ene K. W., Nordberg G., Bergh I., Johansson F. G., Sjöström B. (2008). Postoperative pain management - the influence of surgical ward nurses. *Journal of Clinical Nursing*.

[19] Rafati F., Soltaninejad M., Aflatoonian M., Mashayekhi F. (2016). Postoperative pain: management and documentation by Iranian nurses. *Materia Socio Medica*.

[20] Shoqirat N., Mahasneh D., Dardas L., Singh C., Khresheh R. (2019). Nursing documentation of postoperative pain management. *Journal of Nursing Care Quality*.

[21] Layman Young J., Horton F. M., Davidhizar R. (2006). Nursing attitudes and beliefs in pain assessment and management. *Journal of Advanced Nursing*.

